# Supplementary material for: Dual mTOR/DNA-PK Inhibitor CC-115 Induces Cell Death in Melanoma Cells and Has Radiosensitizing Potential
Source: Int J Mol Sci. 2020 Dec 7;21(23):9321. doi: 10.3390/ijms21239321 (PMC7730287; doi:10.3390/ijms21239321)
Supplement: Supplementary file 1 [file ijms-21-09321-s001.pdf]

International Journal of *Molecular Sciences*

MDPI

Supplementary Material

1 Supplementary Figures

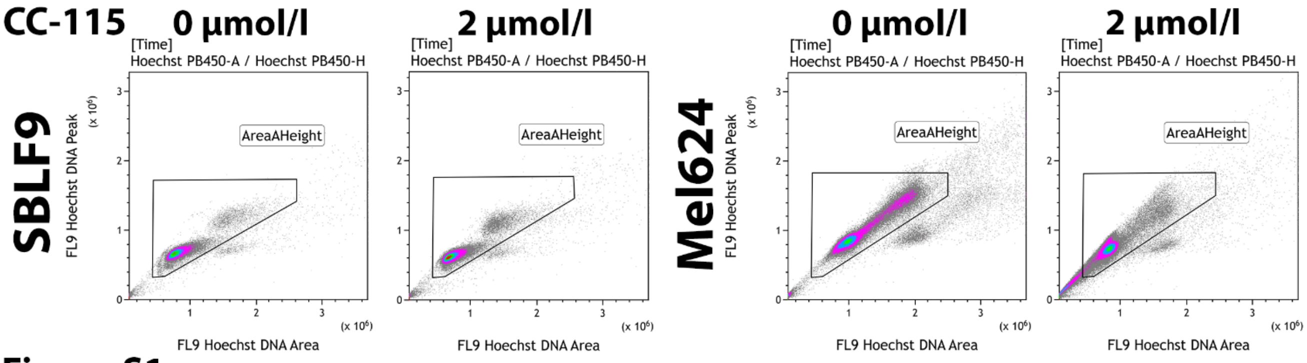

Figure S1

Figure S1: Gating strategy for cell cycle distribution

Flow cytometry was used for cell cycle analysis by Hoechst staining. Examples of representative data are displayed for healthy-donor skin fibroblasts (SBLF9) and melanoma cells (Mel624) for control and treatment with 2 µmol/l CC-115. Graphs show DNA content Area plotted versus DNA content Height. Thereby doublets were excluded.
